# Knowledge and perception regarding molar incisor hypomineralisation among dental students and dental hygienist students in Spain: a cross-sectional study

**DOI:** 10.1186/s12903-024-04074-3

**Published:** 2024-03-02

**Authors:** Vallivana Tarazona-Valero, José Manuel Almerich-Silla, José Enrique Iranzo-Cortés, José Carmelo Ortolá-Siscar, Teresa Almerich-Torres

**Affiliations:** Departament d’Estomatologia, Facultat de Medicina i Odontologia, Universitat de València, Gascó Oliag, 1, Valencia, 46010 Spain

**Keywords:** Molar incisor hypomineralisation, MIH, Knowledge, Perception, Dental students, Dental hygienist students

## Abstract

**Background:**

Molar incisor hypomineralisation (MIH) has a high prevalence in the Spanish pediatric population and is a precursor of carious lesions in teeth in which it is present. Although this pathology is included in the curricula of the Degree in Dentistry and the Training Cycle in Oral Hygiene in our country, the contents currently taught seem to be insufficient in relation to the level of knowledge that we have today about this condition.

**Methods:**

A digital questionnaire of 18 questions was sent to a sample of 448 students attending the 4th and 5th year of the Degree in Dentistry and 2nd year of the Training Cycle in Oral Hygiene from different universities and vocational training centers in the Valencian Community. Descriptive and multivariate statistical analysis of the data was subsequently performed.

**Results:**

Of the 290 questionnaires that were obtained, 53.8% were from students attending the 2nd year of a training course in oral hygiene and 46.2% were from students pursuing a degree in dentistry. Most of the respondents had heard about MIH (75.2%), mainly through master classes. However, most students had difficulties distinguishing MIH lesions from other lesions (58.3%). The degree of knowledge about MIH was greater among dental students in all the aspects evaluated: prevalence, diagnosis, prevention, and treatment. Of all the students, 83.8% were interested in increasing their training on MIH, especially in the areas of diagnosis and treatment.

**Conclusion:**

The results of the present study justify the need to expand the content on MIH, both theoretical and practical, in the educational curricula of the Degree in Dentistry and Integrated Vocational Training Centers in Spain.

## Background

The molar incisor hypomineralisation (MIH) pathology was named and agreed upon in 2001 [1]. The pathognomonic signs are described as defined lesions, asymmetric, with qualitative defects in calcification and maturation of ameloblasts, with a reduction in density and mineral content with respect to healthy teeth, and with the presence of porous enamel whose etching pattern is abnormal [2].

The aetiology of MIH is inconclusive but may be the result of environmental–gene interactions. Different hypotheses have been described such as perinatal hypoxia, and other hypoxia-related perinatal problems, appear to multiply the risk of having MIH. Fever or antibiotic use, which may be considered as consequences to infant and childhood illnesses, have also been implicated. The role of genetic predisposition and epigenetics can be considered as the key information currently missing to truly understand the etiology of MIH [3]. Its differential diagnosis has been related to other pathologies of the dental tissues such as amelogenesis imperfecta, fluorosis, white spot, traumatic hypomineralization among others [4–9]. 

MIH-affected teeth need between 5 and 10 times more dental treatments than healthy teeth, as indicated by the *treatment need index for MIH* (MIH TNI) [10]. Additionally, it has been observed that MIH is a risk factor of caries [11]. 

The level of knowledge, perception, and clinical management of MIH by professionals is an important aspect to consider, given the high prevalence of this pathology in the Spanish pediatric population, 21.8% in 2013 [12].

The MIH condition is currently included in the university curricula of dentistry degrees and implies that both students and dentists who have recently graduated have some knowledge of this pathology compared to those who have graduated 10 or more years ago [5]. The term MIH also appears in the proposal of Spanish Curriculum of Cariology for undergraduate studies in Dentistry [13]. The four dental schools of the Valencian region include at least one topic on MIH in their curricula, normally in pediatric dentistry subjects However, even so, the content currently taught is still insufficient, since preventive, diagnostic and therapeutic approaches are still a pending task to be deepened and disseminated through the creation of national action guides [4, 9, 14].

If we look at the level of training or specialization of dentists, general dentists without specialization have a lower level of knowledge about this pathology than pediatric dentists [5–7, 15, 16]. Dental hygienists, within their training curricula, are also lacking in the study of MIH as a pathology and in turn, its exploration, prevention, and treatment options [17].

In Spain, the regulation governing oral health professions is of the highest legislative rank [18]. This law applies to dentists, as well as other professionals involved in oral health, including dental hygienists and dental technicians. The development of this law’s regulations establishes that dental hygienists perform various functions in the field of public health. These functions include data collection on the state of the oral cavity for clinical or epidemiological use, individual or collective health education, control of preventive measures carried out by patients, or oral health examinations of the community [19].

This study presents a novel approach by including surveys on oral hygiene students. Regarding the clinical relevance, expanding the contents about MIH in the educational curricula of Dentistry Degree and Dental hygiene studies, and creating standardized acting guidelines for molar incisor hypomineralisation can improve the diagnostic and clinical management of MIH by future professionals.

The aim of this study is to evaluate and compare the knowledge levels of dental students and dental hygiene students in the diagnosis and treatment of MIH through online questionnaires. It has been hypothesized that students in the final years of the dentistry degree would have a higher level of knowledge of MIH than students in the second year of the vocational training cycle in oral hygiene. The secondary objectives of this study are to evaluate the need for reviewing curricular contents related to MIH in the dentistry degree and oral hygiene vocational training cycle, and to determine the necessity of creating standardized acting guidelines for improving clinical management of MIH cases.

## Methods

This cross-sectional observational study was carried out at the Stomatology Department of the University of Valencia with the approval of the Human Research Ethics Committee of the University of Valencia (ref. XE182RDB35E4FE9D) in compliance with the ethical principles of the Declaration of Helsinki and current data protection regulations.

To increase the quality of the study, the STROBE Initiative Statement [20] was followed, observing the different items contained in this guide for the preparation and publication of observational studies within each section of the study (title and summary, introduction, methodology, results, and discussion).

### Sample

A calculation was made of the required sample size. It was estimated that, for a population of 250 students attending the 4th and 5th year of the degree in dentistry in the four universities of the Valencian Community, (one public and three private, with one private university declining to participate), a sample of 150 surveys would be sufficiently representative, with a confidence level of 95% and an error of 5%; in the case of students in the vocational training cycle in oral hygiene, it was estimated that, for a population of 400 students attending the 2nd year of the vocational training cycle, 184 surveys would be sufficient, with a confidence level of 95% and an error of 5%. The surveys were conducted in 14 dental hygiene schools, 3 public and 11 private, of which 3 public and 7 private schools agreed to participate. The calculations were performed using the questionpro.com sample size calculator.

The inclusion criteria for participation in the study were as follows: to be a student attending the fourth and/or fifth year of the degree in dentistry at both public and private universities based in the Valencian Community or to be a student attending the second year of the training cycle in oral hygiene at public and private integrated vocational training centers and secondary education institutes in the Valencian Community; only surveys from students who provided informed consent were accepted.

### Data collection

The information on the level of knowledge of MIH in students of the degree in dentistry and vocational training cycle in oral hygiene was obtained by means of an online questionnaire of 18 close-ended questions (some with dichotomous answers, others with exclusive options, and others with multiple answers) that asked about demographic data, gender, age, academic institution, type of studies pursued and levels of knowledge of MIH. The questionnaire was composed of the most frequently asked questions in the literature review articles [5, 6, 14, 15, 17, 21]. The questions, originally in English, were translated by two researchers into European Spanish. The Spanish questionnaire was then back-translated by a native English-speaking translator and the researchers subsequently checked that the back-translation matched the original English wording of the questions. The final questionnaire was inserted into the Limesurvey® platform, licensed by the University of Valencia, with full guarantees regarding the protection of the stored data, thus converting it into an online response questionnaire.

With the final version of the questionnaire [see additional file 1], a pilot test was carried out to check that the platform worked properly and then the questionnaire was disseminated to all students through a virtual link published on the social networks and/or digital teaching platforms of the centers or universities. Those who were interested in participating had to follow the electronic link to the questionnaire, read the presentation information, and give their consent to participate before accessing the questionnaire.

The pilot test was carried out with a sample of 15 students from the oral hygiene training cycle and another 15 students from the fifth year of the degree in dentistry to ensure the readability and comprehension of the questions in the questionnaire. Once the pilot test was completed, the questionnaire was sent to the educational and university centers of the Valencia Community through an e-mail explaining the type of study, mentioning that participation was voluntary and anonymous, and requesting their authorization to disseminate the survey among their students. The questionnaire was available for completion between March 1 and May 1, 2022. Once the questionnaires were completed, statistical analysis of the data was performed. The responses obtained were stored in a database in the Microsoft® Excel® program.

### Data analysis

A simple univariate and multivariate statistical analysis were performed with the IBM® SPSS statistical program (version v 24) with means and proportions.

At the same time, an analytical statistical analysis was performed by groups, according to the type of studies completed (degree in dentistry and vocational training) and subgroups of studies completed (4th year of degree, 5th year of degree and vocational training). This permits the calculation of the chi-square with its p value or asymptotic bilateral significance, together with the symmetrical measure of Cramer’s V, indicating the association strength of the independent variables’ associations tested in the chi-square.

## Results

The questionnaire was sent to 448 students, the total number of students (Dental students DS and Dental Hygiene students DHS) from the schools that agreed to participate in this study. A total of 316 responses were received, but only 290 questionnaires were fully completed and considered valid. The response rate was 70.5%, calculated as the percentage of the received responses out of the 448 questionnaires sent; the effectiveness rate of the questionnaire was 91.8%.

From the valid responses, 132 students belonged to public schools, and 158 students belonged to private schools. Four public schools, including one in Dentistry, and three in the vocational training cycle for oral hygiene, as well as nine private schools, including two in Dentistry and seven in Dental Hygiene, agreed to participate in the survey.

Regarding the type of studies, 134 responses were obtained from students of the degree in dentistry and 156 from students of the vocational training cycle in oral hygiene. The sociodemographic data are presented below (Table 1).

**Table 1:** Sociodemographic characteristics

GENDER	FEMALE	241 (83. 1%)
	MALE	49 (16. 9%)
AGE	< 20 years	58 (20%)
	20–30 years	189 (65.2%)
	> 30 years	43 (14.8%)
TYPE OF CENTER	PUBLIC	132 (45.5%)
	PRIVATE	158 (54.5%)
GROUP OF STUDIES	DS-4	49 (16.9%)
	DS-5	85 (29.3%)
	DHS	156 (53.8%)

DS-4: dental student 4th year; DS-5: dental student 5th year; DHS: dental hygienist student.

Of the 290 responses to the questionnaire, 75.2% of the total number of students surveyed had heard of MIH compared to 24.8% who had not. Referred details to the knowledge and treatment options of MIH among students in the 4th and 5th years of the degree in dentistry and in the 2nd year of the training cycle in oral hygiene are presented in Table 2 in a univariate and multivariate model adjusted by gender, type of center and group of studies.

**Table 2:** Knowledge, perception and treatment options of MIH. Univariate analysis between DS (Dental students) and DHS (Dental Hygiene students). Multivariate analysis for Gender (female/male), type of center (center of study public/private) and Group of studies. Q#: Question number of questionnaire. (*) Statistically significant at 95%.

		Univariate			Multivariate		
Q#	KNOWLEDGE	DS = 134 *n* (%)	DHS = 156 *n* (%)	*p* value	Gender *p* value	Type of Center *p* value	Group of studies *p* value
Q5	Have you heard of Molar Incisor Hypomineralization (MIH)?						
	-Yes	130 (97%)	88 (56.4%)	< 0.001*	0.513	0.005*	0.000*
Q6	Through what source/s have you heard about MIH?						
	-Scientific publications	55 (41%)	22 (14.1%)	< 0.001*	0.495	0.258	0.000*
	-Master classes	103 (76.9%)	45 (28.8%)	< 0.001*	0.239	0.002*	0.000*
	-Conferences and/or courses	33 (24.6%)	10 (6.4%)	< 0.001*	0.272	0.268	0.001*
	-Information brochures	7 (5.2%)	4 (2.6%)	0.237	0.394	0.226	0.280
	-Internet	52 (38.8%)	37 (23.7%)	0.005*	0.567	0.445	0.006*
	-Textbooks	49 (36.6%)	23 (14.7%)	< 0.001*	0.505	0.693	0.000*
	-Clinical practice	93 (69.4%)	22 (14.1%)	< 0.001*	0.773	0.760	0.000*
	-Others	38 (28.4%)	26 (16.7%)	0.017*	0.217	0.872	0.083
Q7	Do you know the clinical features of MIH?						
	-Yes	127 (94.8%)	73 (46.8%)	< 0.001*	0.114	0.026*	0.000*
Q8	Which teeth do you think are most frequently affected?						
	-Permanent first molars and permanent incisors	106 (79.1%)	81 (51.9%)	< 0.001*	0.762	0.564	0.000*
	-Permanent first molars and incisors and temporary second molars	25 (18.7%)	37 (23.7%)	0.295	0.511	0.002*	0.106
	-I do not know/not sure	4 (3%)	43 (27.6%)	< 0.001*	0.112	0.010*	0.000*
Q9	Do you have difficulty distinguishing the MIH lesion from other lesions or malformations of dental tissues?						
	-Yes	64 (47.8%)	102 (65.4%)	0.002*	0.617	0.120	0.005*
Q10	With which ones do you have difficulty distinguishing MIH?						
	-Dental fluorosis	21 (15.7%)	34 (20.7%)	0.185	0.243	0.829	0.313
	-Enamel hypoplasia	66 (49.3%)	43 (27.6%)	< 0.001*	0.885	0.483	0.000*
	-Amelogenesis imperfecta	39 (29.1%)	27 (17.3%)	0.017*	0.526	0.855	0.072
	-Dentinogenesis imperfecta	13 (9.7%)	13 (8.3%)	0.684	0.690	0.378	0.851
	-Caries	15 (11.2%)	4 (2.6%)	0.003*	0.924	0.233	0.020*
	-Other	3 (2.2%)	24 (15.4%)	< 0.001*	0.421	0.487	0.010*
Q11	What factors do you think are involved in the etiology of MIH?						
	-Genetic factors	98 (73.1%)	99 (63.5%)	0.078	0.181	0.689	0.166
	-Chronic diseases of the mother during pregnancy	61 (45.5%)	34 (21.8%)	< 0.001*	0.076	0.855	0.000*
	-Chronic diseases in the child	42 (31.3%)	30 (19.2%)	0.017*	0.115	0.060	0.001*
	-Antibiotics/drugs taken by the mother during pregnancy	52 (38.8%)	40 (25.6%)	0.016*	0.467	0.282	0.047*
	- Antibiotics/drugs taken by the child	27 (20.1%)	23 (14.7%)	0.224	0.490	0.890	0.074
	-Environmental pollutants	34 (25.4%)	15 (9.6%)	0.001*	0.178	0.037*	0.005*
	-Acute illnesses affecting the mother during pregnancy	64 (47.8%)	31 (19.9%)	< 0.001*	0.587	0.479	0.000*
	- Acute illnesses affecting the child	45 (33.6%)	17 (10.9%)	< 0.001*	0.199	0.985	0.000*
	-Exposure to fluoride	8 (6%)	26 (16.7%)	0.005*	0.725	0.209	0.047*
	-None	2 (1.5%)	2 (1.3%)	0.878	0.998	0.288	0.837
	-Other	5 (3.7%)	21 (13.5%)	0.004*	0.815	0.908	0.015*
Q12	What do you think is the prevalence of MIH in Spain?						
	< 5%	9 (6.7%)	7 (4.5%)	0.407	0.016*	0.426	0.792
	5–10%	39 (29.1%)	38 (24.4%)	0.362	0.039*	0.394	0.043*
	10–20%	37 (27.6%)	32 (20.5%)	0.157	0.212	0.001*	0.347
	> 20%	18 (13.4%)	4 (2.6%)	< 0.001*	0.632	0.887	0.002*
	Not sure	36 (26.9%)	73 (46.8%)	< 0.001*	0.413	0.067	0.007*
Q13	Do you think the pattern of caries in teeth with MIH is different from the pattern of normal caries?						
	Yes	105 (78.4%)	83 (53.2%)	< 0.001*	0.636	0.072	0.000*
	No	22 (16.4%)	18 (11.5%)	0.230	0.286	0.823	0.030*
	Not sure	7 (5.2%)	52 (33.3%)	< 0.001*	0.486	0.171	0.000*
Q14	How confident do you feel about diagnosing a tooth with MIH?						
	-Confident	84 (62.7%)	37 (23.7%)	< 0.001*	0.228	0.449	0.000*
Q15	Would you like to receive more training on MIH in your career/training content?						
	Yes	111 (82.8%)	132 (84.6%)	0.682	0.746	0.269	0.944
Q16	On what aspects of MIH do you think you need to complete your training?						
	-Diagnosis	72 (53.7%)	93 (59.6%)	0.313	0.906	0.492	0.299
	-Etiology	44 (32.8%)	81 (51.9%)	0.001*	0.964	0.689	0.020*
	-Treatment	83 (61.9%)	83 (53.2%)	0.134	0.408	0.846	0.635
	Others: prevention, long-term prognosis, etc.	61 (45.5%)	90 (57.7%)	0.039*	0.910	0.833	0.081
	**TREATMENT OPTIONS**						
	**NONOPERATIVE AND OPERATIVE**						
Q17	What preventive (nonoperative) treatment would you apply to MIH lesions without enamel fracture?						
	-Dental mousse with Recaldent	16 (11.9%)	19 (12.2%)	0.950	0.910	0.052	0.471
	-Dental mousse with Recaldent and fluoride	72(53.7%)	24 (15.4%)	< 0.001*	0.172	0.968	0.000*
	-Professional application of high concentration fluoride varnish (22.600 ppm)	94 (70.1%)	47 (30.1%)	< 0.001*	0.492	0.152	0.000*
	-Professional application of chlorhexidine varnish.	3 (2.2%)	12 (7.7%)	0.037*	0.620	0.363	0.994
	-Sealing of pits and fissures.	49 (36.6%)	36 (23.1%)	0.012*	0.711	0.792	0.008*
	-Application of silver diamine fluoride.	10 (7.5%)	9 (5.8%)	0.561	0.093	0.036*	0.970
	-Others.	3 (2.2%)	18 (11.5%)	0.002*	0.261	0.295	0.149
Q18	What operative treatment would you apply to teeth affected by MIH with enamel fracture?						
	-Microabrasion	8 (6%)	11 (7.1%)	0.711	0.629	0.354	0.372
	-Resin infiltration.	0 (0%)	1 (0.6%)	0.353	--	--	--
	-Conventional glass ionomer	33 (24.6%)	31 (19.9%)	0.330	0.790	0.107	0.086
	-Resin-modified glass ionomer	63 (47%)	74 (47.4%)	0.943	0.543	0.669	0.750
	-Composite filling	66 (49.3%)	70 (44.9%)	0.456	0.568	0.011*	0.032*
	-Silver amalgam filling	1 (0.7%)	3 (1.9%)	0.392	--	--	--
	-Preformed crowns	37 (27.6%)	45 (28.8%)	0.816	0.810	0.112	0.026*
	-Extraction	8 (6%)	5 (3.2%)	0.257	0.904	0.228	0.236

### Knowledge

Regarding knowledge about MIH, DS-4 and DS-5 have higher knowledge than DHS (OR = 40.0 CI 5.285-303.286 and OR 24.3 CI 7.015–84.358 respectively), while students from private centers have heard more frequently about MIH than those from public centers (OR = 2.49 CI 1.317-4.700). DS reported receiving more information on MIH from scientific publications, master classes, conferences and/or courses, Internet, textbooks, and clinical practice than DHS. Students from private centers reported receiving more information from master classes than those from public centers (OR = 2.404 CI 1.3683–4.242). Private center students and DS report greater knowledge of MIH than public schools and DHS, respectively. DS recognize, with a statistically significant difference, the involvement of the first permanent molars and permanent incisors, with respect to DHS, and the students from private centers add the involvement of the second primary molars (OR = 2.685 CI 1.435–5.022). In general, DS report less difficulty in distinguishing the MIH lesion from other lesions or malformations of dental tissues. Specifically, DS recognize a greater likelihood of confusing the diagnosis of MIH with enamel hypoplasia and caries.

Regarding the factors involved in the etiology of MIH, DS cite in higher proportion, compared to DHS, chronic diseases of the mother during pregnancy, chronic diseases of the child, antibiotics/drugs taken by the mother during pregnancy, antibiotics/drugs taken by the child, environmental pollutants, acute diseases affecting the mother during pregnancy, and acute diseases affecting the child. DHS cite fluoride exposure as an etiological factor in higher proportion (OR = 0.334 CI 0.117–0.954).

Only the DS-5 recognized in a higher proportion than DHS that the prevalence of MIH in the Valencian Community is higher than 20% (OR 7.255 CI 2.166–24.305).

DS are more in agreement with the differentiation between caries in MIH teeth with respect to normal caries patterns, while DHS are more hesitant about it. Regarding the diagnosis of MIH, most students had difficulty distinguishing MIH lesions from other lesions (58.3%), but DS are much more confident.

In both surveyed groups, DS and DHS, 83.8% of students expressed interest in completing training in various aspects of MIH. DHS showed a greater interest in receiving training on the etiology of MIH.

### Nonoperative treatment

The non-surgical treatment options for MIH lesions without enamel fracture preferred by DS were dental mousse with Recaldent® and fluoride, professional application of high concentration fluoride varnish (22,600 ppm), and pit and fissure sealants. Students in private schools showed a greater preference for silver diamine fluoride application compared to those in public schools.

### Operative treatment

Regarding the recommended surgical treatment for MIH lesions with enamel fracture, all students preferred the use of resin-modified glass ionomer and composite filling. There were no differences between DS and DHS, although DS-4 showed a greater preference than DHS in the use of composite and preformed crowns. Students from private centers also favored the use of composite fillings more than those from public centers (OR = 1.860 CI 1.150–3.006).

## Discussion

MIH currently represents an important problem in oral health, given its increasing prevalence and early onset. However, it has been shown that there are limitations in the knowledge of this entity not only among dentists, but also among dental students and other professionals in the field, such as dental hygienists [5, 6, 17]. In our country, dental hygienists possess skills in dental exploration and in the performance of non-invasive preventive treatments, considered non-operative treatment of dental caries [13]. Therefore, we evaluated their knowledge of this pathology.

This study defends the hypothesis that students in the final years of the degree in dentistry have a higher level of knowledge on diagnosis and treatment of MIH than students in the 2nd year of the vocational training cycle in oral hygiene. In total we obtained 290 valid responses to our online questionnaire. Other cross-sectional studies on MIH with similar designs to ours have obtained similar participation ranges: 255 responses out of 588 students [7] and 230 responses [8], and others have obtained much lower response rates than our study [4]. We surveyed students in the 4th and 5th years, as they receive their first knowledge of this pathology in the fourth year; other studies collected responses only from students in the last year of the Dentistry degree [6, 14, 16, 21].

In reference to the level of knowledge about MIH pathology by the students, in our study most of the students (75.2%) had heard about MIH at some point. In other recent studies, students also claimed to know about this pathology in high percentages: 80% [16], 95% [6] or 98.6% [5]. The multivariate analysis shows us that students from private centers declare that they have heard more frequently about the MIH; however, regardless of the type of study center, DS declare that they know the pathology better than the DHS. The main source of information for the students of the vocational training cycle in oral hygiene was the master classes (28.8%), followed by the Internet (23.7%) and others (16.7%). The students of the degree in dentistry also stated that they had heard of the entity mainly through master classes (76.9%), clinical practice (69.4%), or scientific publications (41%), a source that was barely recognized by the students of the formative cycle (14.1%). In the study by Elhennawy et al. conducted in 2020 [21], the students also stated that they had learned about MIH mainly through lectures (master classes), as in our study. In the study by Liu et al. 2022 [16] most of the students reported having acquired knowledge about MIH through textbooks and specialized journals, master classes and social platforms such as blogs specialized in the subject. Silva et al. 2016 [14], on the other hand, state that specialized publications or journals and continuing professional education were the main sources of information among the surveyed dentists.

According to Garcia-Margarit et al. 2014 [12], the prevalence of MIH in the pediatric population of the Valencian Community exceeded 20% in 2014. When Valencian students were asked about the data on the prevalence of MIH in Spain, 46.8% of dental hygiene students stated that they were not sure what this prevalence was, and 2.6% stated that it was higher than 20%. Only the 13.4% students of the degree in Dentistry knew that the prevalence is higher than 20% in Spain and the multivariate analysis shows us that only DS-5 have better knowledge of the current prevalence in our community than the DHS (OR 7.255).

When asked about a differential diagnosis of MIH with other alterations of the hard tissues of the tooth, the vocational training students indicated enamel hypoplasia (27.6%) as the first option followed by dental fluorosis (20.7%); the dental students also indicated enamel hypoplasia as the primary option (49.3%), followed by amelogenesis imperfecta (29.1%). These two entities, amelogenesis imperfecta and enamel hypoplasia, were also identified as the most difficult pathologies to distinguish from MIH among Swiss students [5]. In a study among dentists in Spain, [4], respondents noted that the differentiation of some forms of MIH from enamel hypoplasia could be confusing. In Germany, according to Elhennawy et al. 2021 [21], 20% of students reported difficulty in making a differential diagnosis of MIH with other enamel pathologies, agreeing with Kalkani et al. 2015 [9], in that British dentists had difficulty distinguishing MIH from other conditions, choosing amelogenesis imperfecta as the most difficult to differentiate from MIH.

In terms of etiology, most of the vocational training students pointed to genetic factors as the main cause (63.5%) followed by the use of antibiotics/drugs by the mother during pregnancy (25.6%) and chronic diseases of the mother during pregnancy (21.8%); dental students agreed that genetic factors are the predominant cause for the development of MIH (73.1%), followed by acute diseases affecting the mother during pregnancy (47.8%) and chronic diseases affecting the mother during pregnancy (45.5%). The same occurs in the studies of Hamza et al., 2021, Silva et al., 2016 and Skaare et al., 2021 [5, 14, 17] where genetic factors, antibiotic and drug treatment, and diseases in both mothers and children were identified as the predominant etiological causes. In a study conducted on Spanish dentists, the majority (42.8%) attributed MIH to chronic diseases in the mother, antibiotic use by the mother during pregnancy (24.8%) and environmental factors (11.6%) [4].

In our study, the differences in diagnostic confidence between the 3 groups of students surveyed (training cycle, 4th year dental student and 5th year dental student) were statistically significant (*p* < 0.001*). Bekes et al. 2021 [6] also found differences in the level of diagnostic confidence between students of different levels. Among 11th-semester dental students, only 5% claimed to be confident diagnosing MIH, compared to 16.2% of 12th-semester dental students. These results confirm the hypothesis of this study regarding the greater degree of knowledge on diagnosis of MIH among undergraduate students of Dentistry degree compared to the students of a Training cycle of professional training in Oral Hygiene. The fact that hygienists do not perform invasive restorative treatments on patients accentuates the idea that the treatment of MIH is more challenging for graduates than for oral hygiene technicians [17].

Successful nonoperative preventive treatments and operative treatments of MIH have been well described for permanent first molars, classifying them according to the severity of the defect and the age of the patient. However, for treatments in anterior teeth the published evidence is still limited [3]. When we asked the students about the nonoperative treatment of MIH, the professional application of high-concentration fluoride varnish (22,600 ppm) was the most chosen option among both students in the formative cycle (30.1%) and dental students (70.1%) in agreement with the answers obtained in the studies of Gamboa et al. 2018 and Liu et al. 2022 [7, 16]. As a second option, dental students pointed to dental mousse with Recaldent® and fluoride (53.7%), in contrast to the studies of Gamboa et al. 2018 and Liu et al. 2022 [7, 16] where resin-based pit and fissure sealants were the second most chosen option.

Regarding operative treatment, therapeutic options are differentiated based on the affected tooth group. For molars, documented therapeutic options include infiltration of lesions with resins, glass ionomer or resin-modified glass ionomer restorations, preformed stainless steel crowns, and even planned extraction in severe cases for children aged 8 to 10 years, followed by orthodontic treatment. However, a recent randomized clinical trial [22] found that conventional resin-based sealants in permanent first molars have better long-term clinical performance than ionomer sealants with self-etch adhesive. This may be due to etching failure in teeth affected by MIH. For patients over 18 years of age, suggested treatments for incisors include enamel microabrasion, bleaching, Wright’s etch-whiten-seal technique, resin infiltration, composite restorations, and/or composite and porcelain veneers [10].

Dental Hygienist students chose resin-modified glass ionomer restorations (47.4%) as the first option, followed by composite restorations (44.9%) and preformed crowns (28.8%); however, undergraduate Dentistry students chose composite restorations (49.3%) followed by resin-modified glass ionomer (47%) and preformed crowns (27.6%) as the therapeutic options, in accordance with with Gamboa et al. 2018 [7]. In other study [16], preformed crowns were the most common operative treatment. For Gambetta-Tessini et al. 2016 [8], glass ionomer cements and resin-reinforced glass ionomers were chosen as the first therapeutic option. In the case of Serna-Muñoz et al. 2021 study [4], resin-modified glass ionomer cement and composite fillings or preformed crowns were indicated, depending on the durability required by the practitioner or the age of the patient. In the study by Skaare et al. 2021 [17], composite filling was the treatment of first choice, followed by glass ionomer cement and preformed crowns. According to Lygidakis et al. 2022 [3], composite filling, preformed metal crowns and laboratory indirect restorations have high success rates for posterior teeth depending on the severity of the cases, while scheduled extractions are an established alternative in severe cases.

Many of both DHSs and DSs expressed a desire to expand the received knowledge about MIH to their respective degrees. DHSs indicated that they would like to expand their knowledge in all the aspects assessed: diagnosis, prevention, treatment, and etiology. DSs stated their interest above all in treatment, followed by diagnosis, prevention, and, with less interest, etiology. These results agree with similar studies in which students and professionals were also asked this question [4–7, 16, 17]. Literature have shown the importance and need to deepen our knowledge of the etiology of MIH and its diagnosis, prevention, and treatment. The objective is to achieve the ability to manage it through noninvasive or minimally invasive therapies. The clinical competencies of dental hygienists in Spain and many other countries include performing non-operative preventive therapies and assisting dentists in operative treatments of MIH, which is why we thought it would be interesting to measure their knowledge of this pathology. This is a novelty compared to other studies in the literature where only one publication [17], to our knowledge, has included them.

This implies the need to establish a curricular debate and to unify knowledge through the creation of MIH treatment protocols in the training of both dental graduates and senior technicians in oral hygiene [4, 17].

## Limitations

Among the possible limitations and weaknesses of the present study, there is the difficulty of accessing all the groups of students from the different universities, secondary education institutes, and integrated centers of vocational training cycles (CIPFP) of the territory of the Valencian Community, which total 14 centers. Additionally, the management of some centers declined to participate in the study.

## Conclusions

The knowledge level of MIH was higher in all the aspects evaluated (prevalence, diagnosis, prevention, and treatment) among the students of the degree in dentistry compared to the students of the vocational training cycle in oral hygiene.

There is a clear need and desire on the part of the students to broaden both the theoretical and practical knowledge of MIH in the educational curricula of the degree in dentistry and vocational training cycle in oral hygiene. It has been shown that they are not sufficiently updated or widespread.

## Recommendations

The need to unify knowledge has been observed through the creation of standardized protocols that facilitate the application of the same therapies regardless of the region or country where the preventive or therapeutic approach is carried out.

The current educational curriculum for the 2022 Vocational Training Cycle in Oral Hygiene in the Valencian Region does not include the term MIH in its program. Considering the current prevalence of this pathology and the results of this study, it is essential to include it due to the role that dental hygienists play in preventive therapies for MIH.

Prospective studies based on curricular modifications will make it possible to assess the effectiveness of these modifications and their consequences in the training of dentists and hygienists in the management of MIH.

## Data Availability

The datasets used and analyzed during the current study available from the corresponding author on reasonable request.

## Abbreviations

MIH: Molar incisor hypomineralisation
CIPFP: Public integrated vocational training center
SNS: National Health system
DS: dental student
DHS: dental hygienist student
